# Exercise-Mediated Wall Shear Stress Increases Mitochondrial Biogenesis in Vascular Endothelium

**DOI:** 10.1371/journal.pone.0111409

**Published:** 2014-11-06

**Authors:** Boa Kim, Hojun Lee, Keisuke Kawata, Joon-Young Park

**Affiliations:** 1 Department of Kinesiology, Temple University, Philadelphia, Pennsylvania, United States of America; 2 Cardiovascular Research Center, Temple University, Philadelphia, Pennsylvania, United States of America; RIKEN Advanced Science Institute, Japan

## Abstract

**Objective:**

Enhancing structural and functional integrity of mitochondria is an emerging therapeutic option against endothelial dysfunction. In this study, we sought to investigate the effect of fluid shear stress on mitochondrial biogenesis and mitochondrial respiratory function in endothelial cells (ECs) using *in vitro* and *in vivo* complementary studies.

**Methods and Results:**

Human aortic- or umbilical vein-derived ECs were exposed to laminar shear stress (20 dyne/cm^2^) for various durations using a cone-and-plate shear apparatus. We observed significant increases in the expression of key genes related to mitochondrial biogenesis and mitochondrial quality control as well as mtDNA content and mitochondrial mass under the shear stress conditions. Mitochondrial respiratory function was enhanced when cells were intermittently exposed to laminar shear stress for 72 hrs. Also, shear-exposed cells showed diminished glycolysis and decreased mitochondrial membrane potential (Δ*Ψ*m). Likewise, in *in vivo* experiments, mice that were subjected to a voluntary wheel running exercise for 5 weeks showed significantly higher mitochondrial content determined by *en face* staining in the conduit (greater and lesser curvature of the aortic arch and thoracic aorta) and muscle feed (femoral artery) arteries compared to the sedentary control mice. Interestingly, however, the mitochondrial biogenesis was not observed in the mesenteric artery. This region-specific adaptation is likely due to the differential blood flow redistribution during exercise in the different vessel beds.

**Conclusion:**

Taken together, our findings suggest that exercise enhances mitochondrial biogenesis in vascular endothelium through a shear stress-dependent mechanism. Our findings may suggest a novel mitochondrial pathway by which a chronic exercise may be beneficial for vascular function.

## Introduction

Mitochondria are multifunctional organelles. Not only are they metabolic hubs, but they are also involved in other vital cellular processes. In endothelial cells (ECs), the potential physiological role of mitochondria has been somewhat neglected because their energy supply is relatively independent of the mitochondrial respiration, although the accuracy of this notion as it relates to other mitochondrial functions in the cells is unknown. To this end, emerging evidence suggests that mitochondria are essential for maintaining various endothelial homeostasis such as ROS signaling, Ca^2+^ regulation, apoptosis and cell senescence [1]–[9]. Furthermore, mitochondrial dysfunction has appeared to be responsible for the range of cardiovascular diseases intimately related with endothelial dysfunction such as hypertension and atherosclerosis [1], [3], [4], [8]–[13]. Thus, it is imperative to identify an effective intervention to manipulate mitochondrial networks in the endothelium.

The regular practice of physical activity is one of the most effective non-pharmacological interventions improving endothelial dysfunction. During the last two decades, the beneficial effects of exercise on the vascular endothelium have been extensively studied in various aspects of the endothelial function related to endothelium-dependent vasodilatory, anti-inflammatory, anti-thrombotic, and anti-apoptotic endothelial phenotypes [14]–[26]. Whilst exercise-induced uniaxial laminar flow has been thought to be the central signaling mechanism for the endothelial adaptations [27]–[33], a direct impact of this flow pattern on endothelial mitochondrial adaptations *in vivo* is unknown.

Mitochondrial biogenesis is a complex process involving the replication of mitochondrial DNA (mtDNA) and the expression of mitochondrial proteins encoded by both nuclear and mitochondrial genomes. Peroxisome proliferator-activated receptor-γ coactivator-1α (PGC-1α) transactivates nuclear respiratory factor 1 (NRF-1) which, in turn, activates mtDNA transcription factor A (TFAM) that regulates mtDNA transcription and replication. The activation of PGC-1α involves a dual-posttranslational modification involving AMP-activated protein kinase (AMPK) and NAD-dependent protein deacetylase, sirtuin 1 (SIRT1), but the specific regulatory mechanism in ECs remains controversial [34], [35]. p53-inducible ribonucleotide reductase (p53R2) plays a crucial role in a salvage pathway to supply dNTPs for mtDNA synthesis [36]. In addition, upregulation of other mitochondrial contents including respiratory chain complexes and their assembly proteins (i.e., COX IV, SCO1 and SCO2) are also important for preventing dilution of the contents for a successful mitochondrial proliferation. Mitochondrial dynamics plays a crucial role in mitochondrial quality control. Mitochondrial fission is achieved through the action of a set of proteins, including dynamin-related protein, Drp1, and outer-membrane receptor-like protein, Fis1. Mitochondria fusion involves outer mitochondrial membrane proteins, mitofusins 1 and 2 (Mfn1 and Mfn2) and an inner membrane protein Opa1 [37]. Through proper fusion/fission dynamics coordinated with contents amplification, new daughter mitochondria are formed [38].

Recently, potential link between shear stress and mitochondrial biogenesis in ECs has been suggested [39]–[42]. Chen *et al*. reported that laminar flow upregulates the key mitochondrial biogenesis regulators including PGC-1α and SIRT1 as well as the MitoTracker Green signals in shear-exposed ECs [39]. In addition, a study reported that a short-term forced exercise on a motorized treadmill significantly altered mitochondrial dynamic protein profiles in the rat aortic tissues in a NO-dependent fashion [40]. Here, we report that laminar shear stress (LSS) increases mitochondrial biogenesis/dynamics and mtDNA content, and modulates their respiratory function and bioenergetics in human ECs. We also report that chronic voluntary running exercise increases mitochondrial density in the mouse endothelium in a shear stress-dependent manner. Findings from this study will help understand the effects of aerobic exercise-mediated increase in wall shear stress (WSS) on enhancing mitochondrial contents which might be a guide of therapeutic approach for improving cardiovascular health.

## Materials and Methods

### Cell culture and LSS protocol

**Figure 1:**
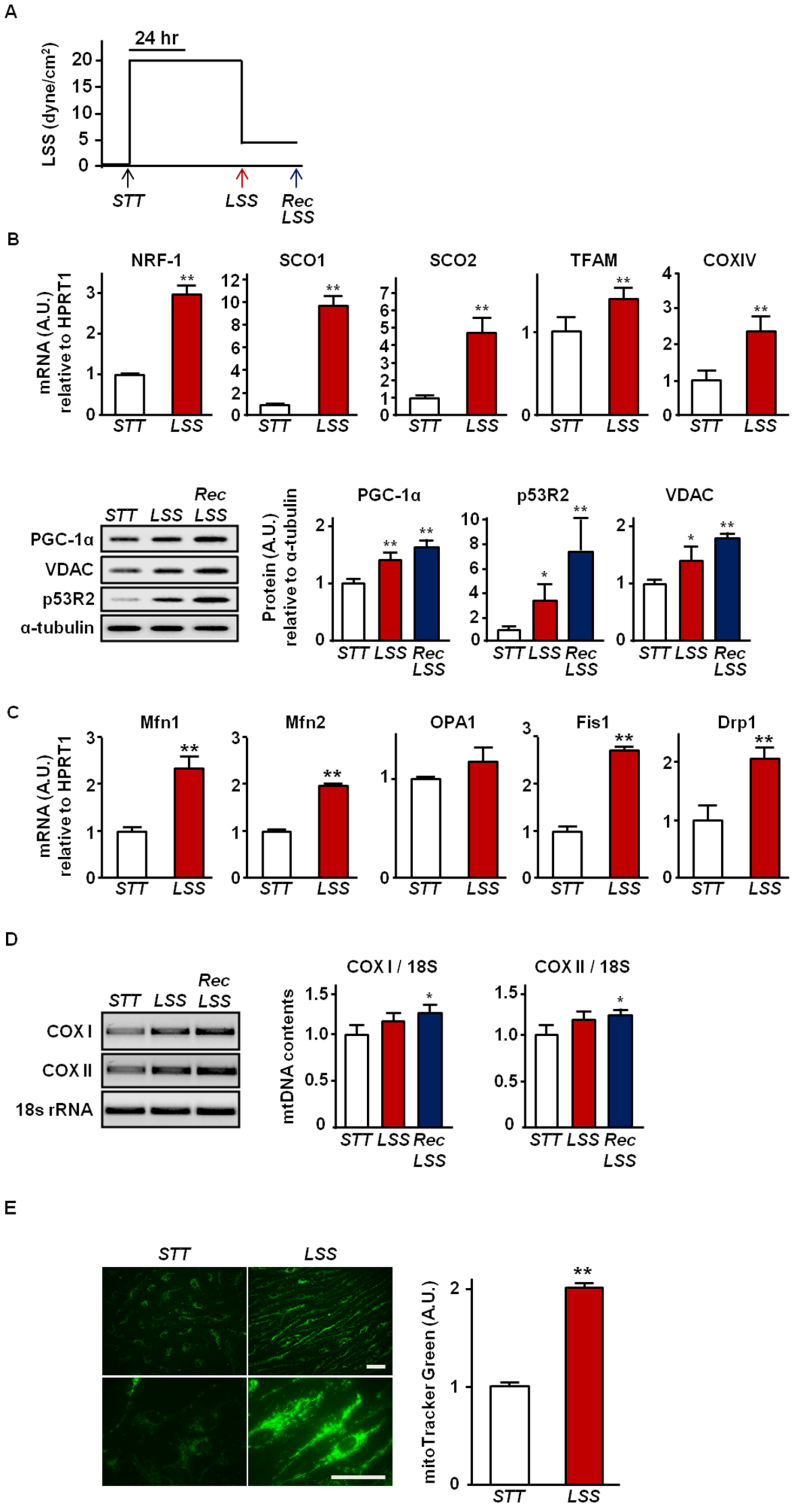
Increased mitochondrial biogenesis markers by LSS in HAECs. (a) An overview of LSS protocol used. HAECs were exposed to exercise-mimicking LSS at 20 dyne/cm^2^ for 48 hrs, and then, recovery (Rec) LSS at 5 dyne/cm^2^ was followed for another 24 hrs. (b) Effect of LSS on the mRNA and protein expression of mitochondrial biogenesis markers. mRNA expression of NRF-1, SCO1, SCO2, TFAM, and COX IV were assessed by real-time PCR and protein contents of PGC-1α, VDAC, and p53R2 were analyzed by western blot. (c) Effect of LSS on the mRNA expression of mitochondrial dynamics markers. mRNA expression of Mfn1, Mfn2, OPA1, Fis1, and Drp1 were assessed by real-time PCR. (d) Effect of LSS on mtDNA contents. Relative mtDNA contents are expressed as a ratio of COX I and II to 18s rRNA. (e) Effect of LSS on mitochondrial mass. Mitochondria were labeled with MitoTracker Green in live HAECs. Representative fluorescence micrographs under STT (left panel) and after 48 hrs of LSS at 20 dyne/cm^2^ (right panel) are shown. Bar  = 50 µm. The MitoTracker Green fluorescence intensities were analyzed using the Image J (NIH) software. All densitometry analyses values are shown as mean ± SE; * *P*<0.05 vs. STT; ** *P*<0.01 vs. STT.

Human aortic ECs (HAECs) and human umbilical vein ECs (HUVECs) (Lonza) were cultured in EGM-2 and M199 medium supplemented with 20% fetal bovine serum and endothelial cell growth supplement, respectively. Cells were exposed to the arterial levels of LSS for various time points by using a cone-and-plate shear system once they reach at 100% confluency. Overview of the LSS protocol is outlined in figure 1A. All experiments with HAECs and HUVECs were conducted between the 3–7 passages.

### Immunoblotting

Cells were washed three times with cold DPBS and lysed in RIPA buffer (10 mM Tris-HCl, 5 mM EDTA, 150 mM NaCl, 1% Triton X-100, 0.1% SDS, 1% Deoxycholate, pH 7.5). Following precipitation of insoluble fraction of the RIPA samples by centrifugation (16,000 g for 15 min at 4°C), supernatants were collected and subjected to Bradford assay to quantify protein concentrations. The resulting protein samples underwent SDS-PAGE and were transferred to Immobilon-P membrane (Millipore). Subsequently, the membrane was blocked with 5% nonfat dry milk in TBST for 20 min at room temperature and incubated overnight with respective primary antibodies. Antibodies were purchased from the following sources: rabbit polyclonal anti-PGC-1α (Novus), mouse monoclonal anti-porin (anti-VDAC) (Invitrogen), goat polyclonal anti-p53R2 (Santa Cruz), rabbit polyclonal anti-AMPKα (Cell signaling), rabbit polyclonal anti-phospho-AMPKα (Cell signaling), mouse monoclonal α-tubulin (Sigma-Aldrich). The membranes were then washed twice in TBST and incubated with HRP-conjugated secondary antibodies for an hour followed by washing three times with TBST. Then, membranes were subjected to standard enhanced chemiluminescence (Thermo Fisher Scientific) method for visualization.

### mRNA isolation, cDNA synthesis, and real-time PCR

mRNAs were isolated using Dynabeads direct kit, and cDNA synthesis were performed on poly-dT magnetic beads by reverse transcription using superscript II (Invitrogen). mRNA expression levels were quantified by real-time PCR using SYBR green fluorescence. Cycle threshold (Ct) values were normalized to the housekeeping gene HPRT1. The primer sequences used are described in Table 1.

**Table 1:** Primer Sequences for Real-Time PCR.

Species	Genes	Primer sequences (5′ – 3′)	
		Sense	Antisense
human	NRF-1	CCAAGTGAATTATTCTGCCG	TGACTGCGCTGTCTGATATCC
	SCO1	GGCACAGCCAGTGCATTCCTGCCTG	GCATCACACTCGTGATCAATATCCTC
	SCO2	GCAGCCTGTCTTCATCACTGTGGACC	CCGCACACTGTCTGAGATCTGCTC
	TFAM	AGCTAAGGGTGATTCACCGC	GCAGAAGTCCATGAGCTGAA
	COX IV	ACGAGCTCATGAAAGTGTTGTG	AATGCGATACAACTCGACTTTCTC
	HPRT1	GACACTGGCAAAACAATGCAG	AGTCTATAGGCTCATAGTGC
	MFN1	AGTAACAGGATTGGCGTCCG	CGTTTCCTCCTATCATGGTCACC
	MFN2	ATGCATCCCCACTTAAGCAC	CCAGAGGGCAGAACTTTGTC
	OPA1	GGCTCTGCAGGCTCGTCTCAAGG	TTCCGCCAGTTGAACGCGTTTACC
	DRP1	CACAGGAGGAGGTGGACAGC	CGCCTCCTTCAGTGCGTGGT
	FIS1	ATGGAGGCCGTGCTGAAC	TCAGGATTTGGACTTGGA
mouse	PGC-1α	ACGGTTTACATGAACACAGCTGC	CTTGTTCGTTCTGTTCAGGTGC
	NRF-1	GAACGCCACCGATTTCACTGTC	CCCTACCACCCACGAATCTGG
	TFAM	CTGATGGGTATGGAGAAGGAGG	CCAACTTCAGCCATCTGCTCTTC
	p53R2	CCAGGTTACCATGGTTGTGG	CCAGTGCACTCAGTAGCTGTG
	SCO1	CTAGCTTAGCACAATAGCAAGGGCAGGCTAC	CCCAGGAATGCAGTTATGACATGACAGCAAAGGCAG
	SCO2	CAGCCTGTCTTCATCACTGTGGA	GACACTGTGGAAGGCAGCTATGTGCC
	TIF	CTGAGGATGTGCTGTCTGGGAA	CCTTTGCCTCCACTTCGGTC

### mtDNA content quantification

Total genomic DNAs were isolated by using the DNeasy kit (QIAGEN) and mtDNA contents were assessed by semi-quantitative PCR. The relative ratio between mitochondrial DNA (COX I; cytochrome c oxidase subunit I, COX II; cytochrome c oxidase subunit II, or ND II; NADH dehydrogenase subunit 2) compared to nuclear DNA (18s rRNA) amount was calculated. Primer sequences were as follows:

COXI (human)

Sense, 5′- CATAGGAGGCTTCATTCACTG – 3′

Antisense, 5′- CAGGTTTATGGAGGGTTCTTC – 3′

COXII (human)

Sense, 5′- CCATAGGGCACCAATGATACTG – 3′

Antisense, 5′- AGTCGGCCTGGGATGGCATC – 3′

NDII (mouse)

Sense, 5′- CCTATCACCCTTGCCATCAT – 3′

Antisense, 5′- GAGGCTGTTGCTTGTGTGAC – 3′

18s rRNA (human and mouse)

Sense, 5′-CTTAGAGGGACAAGTGGCGTTC-3′

Antisense, 5′-CGCTGAGCCAGTCAGTGTAG-3′

### MitoTracker staining

Live HAECs exposed to either static (STT) or LSS were incubated with 200 nM pre-warmed MitoTracker Green FM or MitoTracker Red CMXRos (Molecular Probes) solution at 37°C for 30 min. After removal of the incubation solution, cells were washed three times with pre-warmed PBS and then mounted in Hank's balanced salt solution. For quantitative analyses, more than 100 images per each group were acquired using an epi-fluorescence upright microscope with a 63x objective oil lens. For MitoTracker Green FM staining, excitation/emission wavelengths were set at 470/525 nm (FL filter Set 38, Zeiss), and for MitoTracker Red CMXRos staining, excitation/emission wavelengths were set at 587/647 nm (FL filter Set 64HE). Images were initially acquired using an AxioCam MRm and AxioVision image processing system (Zeiss), and the fluorescence intensities were assessed using Image J software (NIH).

### Mitochondrial respiration

HUVECs were subjected to intermittent LSS at 20 dynes/cm^2^ for up to 72 hours while the STT control group was maintained in the absence of LSS. Cells were subcultured as needed to avoid becoming over-confluent for the duration of experiments. Cells were then harvested and the oxygen consumption was measured using a Clark-type oxygen electrode in complete media. Final oxygen consumption was normalized to the number of cells (nmol O_2_/min/10^8^ cells).

### Lactate production measurement

Lactate concentration in cell culture medium was measured by a colorimetric enzymatic assay according to the manufacturer's instructions (Sigma). Briefly, when cells were grown at ≈80% confluency, cell culture medium was replaced with fresh basal M199 medium. Then, media samples were collected at 12, 24, and 36 hours after incubation and filtered through 10 kDa molecular weight cut-off spin columns (Milipore) before being subjected to lactate assays. Lactate concentration was normalized to corresponding viable cell numbers determined by trypan blue exclusion quantification.

### Microarray analysis

To gain insight into global patterns of metabolic gene expression, microarray analysis was performed. RNA was isolated by using RNeasy kit (QIAGEN). Microarray analysis were performed from STT (n = 4) and LSS (n = 6) exposed HUVECs by using Affymetrix whole-genome arrays containing 45,101 probe sets corresponding to ≈34,000 genes. Heat map was created with Gene-E ver. 3.0.214 (Broad Institute, Inc).

### Ethics statement

This study was carried out in strict accordance with the recommendations and the Guide for the Care and Use of Laboratory Animals of the National Institutes of Health. The protocol was approved by the Temple University Institutional Animal Care and Use Committee (Permit Number: 4159). All sacrifices were performed under isoflurane anesthesia, and all efforts were made to minimize suffering.

### Experimental animals and voluntary wheel exercise

After three days of acclimation period, twenty inbred C57Bl/6J mice were randomly assigned to either sedentary (SED) (n = 10) or voluntary wheel (VW) running exercise (n = 10) group. VW group animals were individually housed in a rat-sized cage with a metal wheel with a diameter of 11.5 cm (Prevue) fitted with digital magnetic counter. SED group animals were singly housed in the same sized cage without the running wheel. All animals were given water and food (Purina chow) ad libitum. VW running exercise began at an age of 8 to10-week-old and continued for 5 weeks.

### Blood vessel isolation

Mice were euthanized two days after the end of 5-weeks of VW exercise period. For the preparation of RNA, protein, and DNA, abdominal aorta was isolated after whole body perfusion with ice-cold PBS at a pressure of approximately 100 mmHg. For *en face* staining, several different regions of blood vessels including aortic arch, thoracic aorta, femoral artery, and mesenteric artery were isolated after the perfusion with ice-cold PBS and a fixative, 2% paraformaldehyde.

### En face immunostaining

Isolated blood vessels were post-fixed at 0.4% paraformaldehyde overnight at room temperature. The vessels were then washed five times with PBS and permeabilized by using 0.3% Triton-X in 2% BSA/PBS. Mitochondrial contents were assessed by using anti-VDAC (1∶100) (Abcam) antibody and Alexafluor488-conjugated anti-rabbit secondary antibody (Invitrogen). EC were identified by co-staining using anti-CD31 (1∶100) (Millipore) antibody conjugated to the Alexafluor647-conjugated anti-hamster secondary antibody (Jackson ImmunoResearch). Primary antibodies were incubated overnight at 4°C with gentle agitation. After rinsing in 2% BSA/PBS, secondary antibodies were incubated for 2 hours at room temperature. Immunostained vessels were placed on slide glass and cut longitudinally and mounted in ProlongGold with DAPI solution (Invitrogen). The fluorescence was analyzed under fluorescence microscope (Axioimager, Zeiss) with 64x oil objective lens.

### Statistics

The results are presented as mean ± SE for a minimum of three independent experiments in triplicate. Depending on how many conditions were compared, either two tailed t-test analysis or one-way ANOVA with the Fisher's least significant difference test was conducted. *P*<0.05 was considered statistically significant for all analyses.

## Results

### LSS enhances mitochondrial biogenesis in human ECs

As shown in figure 1B, we observed that LSS upregulates mRNA and protein expression of key genes that are related to mitochondrial biogenesis in HAECs. mRNA expressions of NRF-1, TFAM, COX IV, SCO1 and SCO2 were significantly increased in the ECs exposed to LSS. As well, protein expressions of PGC1α, p53R2, and VDAC were increased when cells were exposed to LSS. To confirm the LSS-induced increase in mitochondrial biogenesis, we stained HAECs with MitoTracker Green FM, a fluorescence dye which stains mitochondria in a mass-dependent fashion, and observed two-fold increase in mitochondrial mass in LSS-exposed HAECs (Fig. 1E). As shown in figure 1D, mtDNA contents were also significantly increased by LSS. In addition, expression of both profusion (Mfn1 and Mfn2) and profission (Drp1 and Fis1) factors were significantly increased after LSS exposure (Fig. 1C).

**Figure 2:**
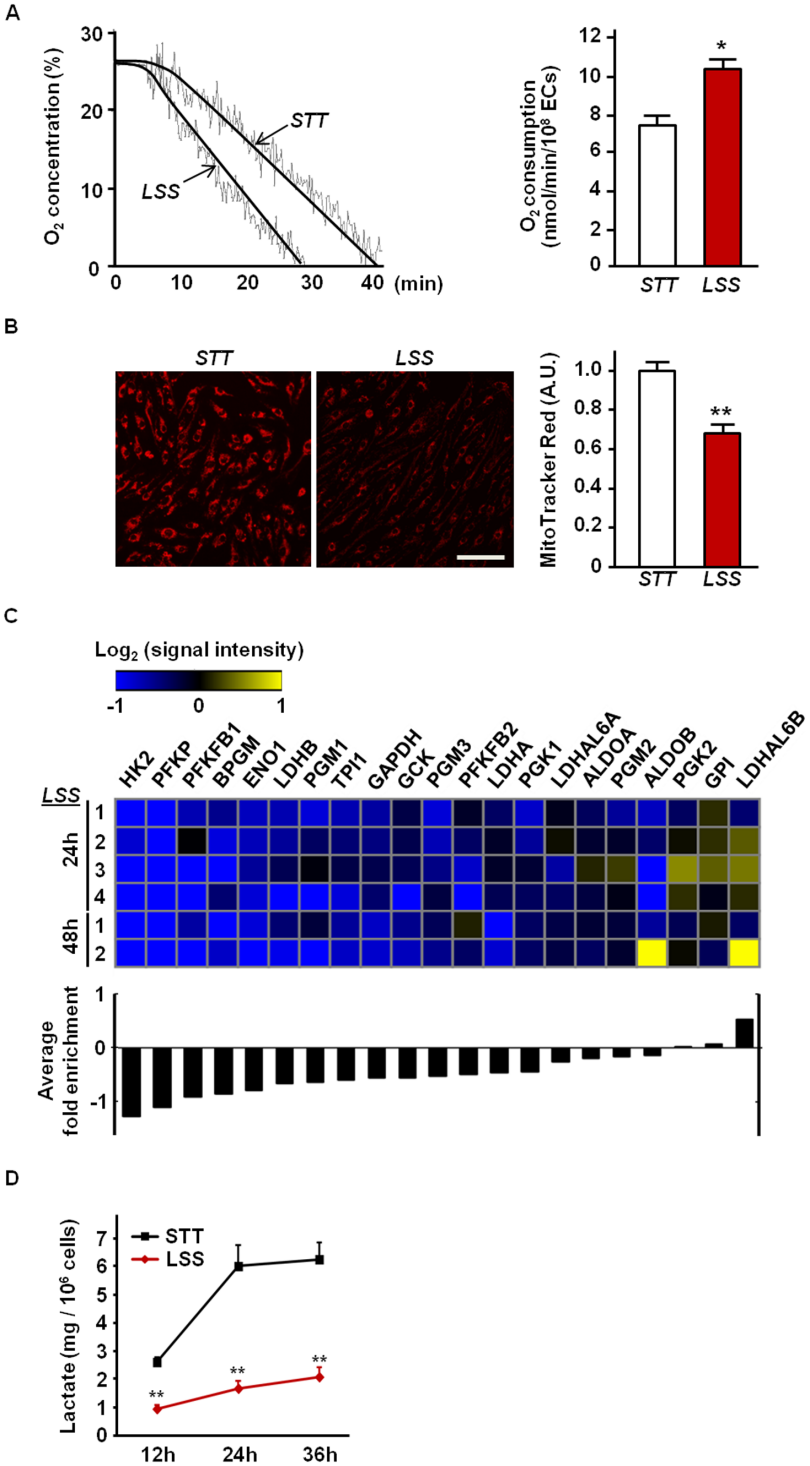
Effect of LSS on endothelial metabolism. (a) Enhanced mitochondrial respiration in LSS-exposed HUVECs. Oxygen consumption of HUVECs was measured after the intermittent LSS exposure for up to 72 hours. Representative strips of the oxygen consumption measured (left panel). Normalized values to the number of cells (right panel). (b) Effect of LSS on Δ*Ψ*m in ECs. Δ*Ψ*m was estimated by using MitoTracker Red CMX Ros. Representative fluorescence micrographs for each condition are shown. Bar  = 100 µm. The fluorescence intensities were analyzed using the Image J (NIH) software. (c) Heat map showing the expression of glycolysis markers by microarray analysis. Genes upregulated are presented in yellow and downregulated are in blue (upper panel). Average fold change of each of those glycolysis markers identified by microarray analysis are shown in a bar graph (lower panel). (d) Lactate concentration measured in cell culture medium at 12, 24, 36 hrs of post LSS or STT. Values were normalized to viable cell number. Data shown as means ± SE; * *P*<0.05 vs. STT; ** *P*<0.01 vs. STT.

Next, we sought to examine whether LSS-induced mitochondrial biogenesis was functionally relevant to the mitochondrial bioenergetic properties. As shown in figure 2A, the rate of oxygen consumption was significantly enhanced in HUVECs after being exposed to LSS for 72 hours. To evaluate a potential occurrence of metabolic shift from glycolytic to aerobic metabolism in these cells, we evaluated cellular lactate production and performed gene expression array experiments on a number of genes related to the glycolytic pathways. Cellular lactate production was significantly suppressed in the LSS-exposed ECs compared to the STT-exposed ECs (Fig. 2D). Moreover, among the twenty-one genes related to glycolysis pathway, the vast majority of genes were down-regulated under LSS (Fig. 2C and Table S1). Notably, these genes include key rate-limiting enzymes for glycolysis such as hexokinase II (HK2) and phospohofructokinase (PFK)-related genes (i.e., PFKFB1, PFKFB2, and PFKP). Mitochondrial membrane potential (Δ*Ψ*m), which was determined by MitoTracker Red CMXRos, was significantly decreased in LSS-exposed ECs compared to STT-exposed ECs (Fig. 2B).

### Five weeks of VW running induces mitochondrial biogenesis in blood vessel and it is mediated by exercise-induced increase in WSS on vascular endothelium

**Figure 3:**
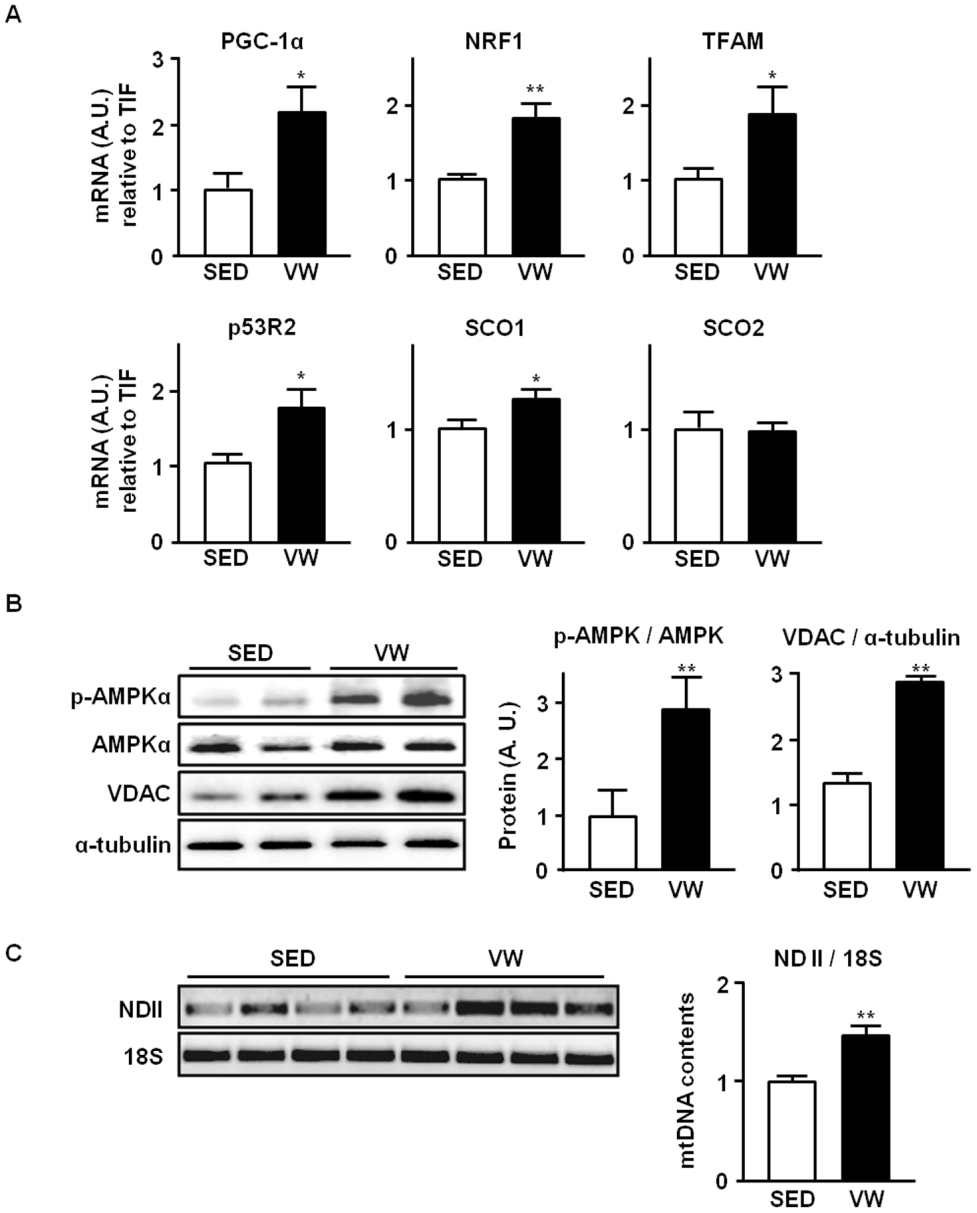
Effect of five weeks of voluntary wheel (VW) exercise on mitochondrial biogenesis markers in mice abdominal aorta (AA). (a) Effect of VW running on mRNA expression of mitochondrial biogenesis markers in AA. mRNA expressions of PGC-1α, NRF1, TFAM, p53R2, SCO1, and SCO2 were examined by real-time PCR. Values were normalized to the level of housekeeping gene, TIF. (b) Effect of VW running on protein expression of mitochondrial biogenesis markers in AA. Tissue extracts of the AA from SED and VW group mice were subjected to western blot. The amount of phosphorylated- AMPKα was normalized by the amount of AMPKα protein. Protein content of mitochondrial biogenesis marker VDAC was also measured. The loading volume was normalized by the expression level of α-tubulin. (c) Effect of VW running on mtDNA content in AA. mtDNA contents were compared in between SED and VW run mice. Relative mtDNA content are expressed as a ratio of NADH dehydrogenase subunit 2 (ND II) to 18s rRNA. All densitometry analyses values are shown as means ± SE. Data shown represent results from a total of 5 mice per group; * *P*<0.05 vs. SED; ** *P*<0.01 vs. SED.

**Figure 4:**
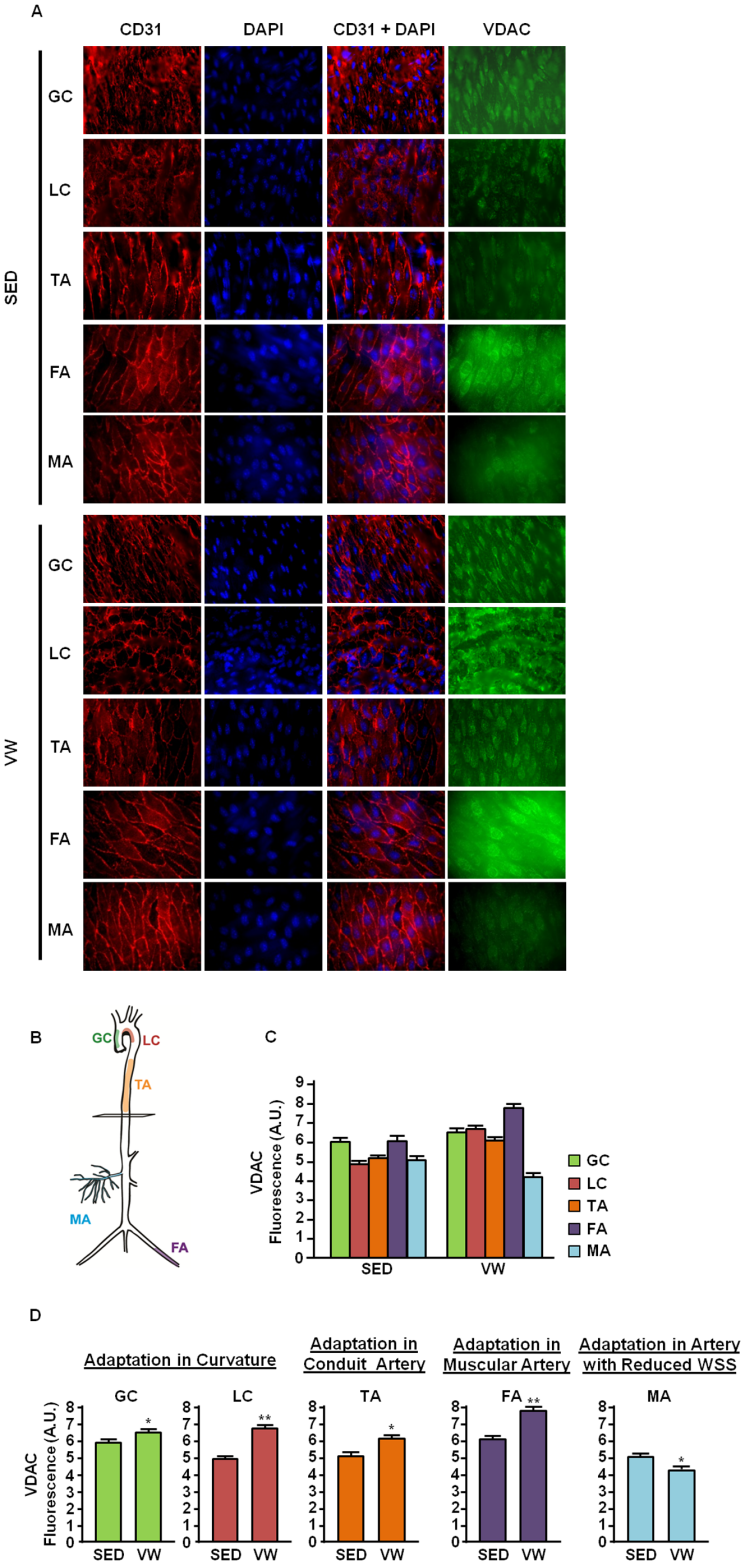
Effect of five weeks of VW running on mitochondrial in mouse endothelium. (A) Representative fluorescence micrographs of *en face* immunostaining. Endothelium of the greater curvature (GC), lesser curvature (LC), thoracic aorta (TA), femoral artery (FA), and mesenteric artery (MA) were stained in the sedentary (SED) and voluntary wheel (VW) run C57BL6 mice. The green fluorescent staining indicates mitochondrial density stained by VDAC, and the red color represents ECs stained by CD31 (an endothelial cell specific marker). Nuclei were counterstained with DAPI. Shown are representative images of *en face* staining labeled. (B) Illustration of mouse arterial tree. (C) Summary of densitometry analysis. Green fluorescence intensities by VDAC staining were analyzed using the Image J (NIH) software. Data shown as mean ± SE; Data shown represent results from a total of 10 mice per group * *P*<0.05 vs. SED. ** *P*<0.01 vs. SED.

Given our observation that LSS is positively related to mitochondrial biogenesis *in vitro*, we hypothesized that exercise-mediated increase in WSS would enhance mitochondrial biogenesis in mouse endothelium. As shown in figure 3A, expressions of genes that are related to mitochondrial biogenesis were analyzed in abdominal aorta isolated from SED and VW group mice. Elevated mRNA expressions of mitochondrial biogenesis markers which include PGC-1α, NRF1, TFAM, p53R2, and SCO1 were observed in VW group mice compared to SED. Also, western blot analysis revealed that phosphorylated AMPKα and VDAC were increased by three-fold in VW group compared to SED (Fig. 3B). Furthermore, greater mtDNA content was found in VW group compared to SED (Fig. 3C). We also hypothesized that differential hemodynamic flow in different vessel beds may lead to distinct responses depending on their geometrical location in the vascular tree. *En face* staining experiment revealed that the level of VDAC protein in greater curvature, lesser curvature, thoracic aorta, and femoral artery was higher in VW group compared to the SED group (Fig 4). VW running elicited greater mitochondrial adaptation in lesser curvature compared to greater curvature. The greatest increase in mitochondrial content was observed in femoral artery. In mesenteric artery, decreased level of mitochondrial content was observed in VW compared to SED group.

## Discussion

Here, we report that LSS enhances mitochondrial biogenesis, mitochondrial dynamics, and mtDNA copy number in primary cultured human ECs. Consistent with these findings, we also demonstrate that voluntary aerobic exercise training increases mitochondrial content in the endothelium in a region-specific fashion. In addition, we found that a long-term shear-exposure is sufficient to improve mitochondrial respiration and to alter substrates metabolism from anaerobic glycolysis to oxidative phosphorylation-dependent mechanisms in ECs. These findings are particularly important because potential metabolic contributions of the endothelial mitochondria have been widely neglected as they are highly glycolytic cells containing relatively small number of mitochondria (only 2-5% of the entire cytoplasmic volume) compared to other energy demanding tissues [43]. Furthermore, studies have demonstrated that, under stress conditions, fatty acids are the major substrate for ATP generation in ECs suggesting an important contribution of mitochondria-dependent metabolism for endothelial homeostasis [44]. To this end, our data suggest that LSS-induced mitochondrial biogenesis may have important implications for preventing endothelial dysfunction although future researches are needed to investigate the effect of LSS (or aerobic exercise training) on the energy metabolism and the substrate utilization in ECs *in vivo*.

In this study, we also demonstrated that a long-term LSS at a physiological level decreased Δ*Ψ*m. This result is consistent with a previous report showing that shear stress induces a decrease in Δ*Ψ*m and an increase in the endogenous ATP [45]. In contrast, a short-term shear stress increases Δ*Ψ*m in ECs suggesting a biphasic temporal response [46]. Δ*Ψ*m is regulated primarily by the balance between electron flux through the respiratory chain (Complexes I, III, and IV), ATP synthesis (coupled respiration), and proton leakage across the inner membrane (uncoupled respiration). Maintenance of Δ*Ψ*m at physiological range is important for regulating mitochondrial ROS production. It has been postulated that there is a U-shaped curve describing the relationship between Δ*Ψ*m and ROS formation [47]. Furthermore, numerous studies have shown that hyperpolarization of the mitochondria (above ∼−140 mV) triggers release of superoxide predominantly at complex III [48]. We observed that UCP2 expression is dramatically elevated under the same shear paradigm used in this study (unpublished data). Combined with evidence that UCP2 inhibits formation of ROS [49], it is plausible that the depolarization of the mitochondria would prevent ROS release. Together, shear stress may improve cellular redox state, at least in part, by modulating Δ*Ψ*m in favor of reduced mitochondrial ROS production which compliment other shear-mediated mechanisms such as a down-regulation of NAD(P)H oxidase activity [50] and an increase in antioxidant system [51], [52].

Different vascular beds are exposed to distinct flow patterns depending on their structural and functional properties. For example, in the aortic arch, greater curvature is exposed to a high-grade unidirectional shear stress where lesser curvature is exposed to a low-grade oscillatory shear stress [53]. Lesser curvature has been shown to be predisposed to atherosclerotic plaque formation. In sedentary mice, we observed that mitochondrial content is higher in the greater curvature compared to the lesser curvature, suggesting a direct correlation between flow pattern and mitochondrial content in the endothelium.

It is well known that a process termed ‘blood redistribution’ occurs during exercise [54], [55]. At rest, only 15–20% of cardiac output is redirected to skeletal muscle and the majority of it goes to the other organs. Once exercise commence, however, 87% of blood is redirected to exercising muscles. Muscle blood flow has been shown to be increased up to 80-fold [56], [57]. Corresponding to this concept, amount of blood extracted by the celiac, mesenteric, and renal arteries is decreased during exercise [27], [54], [55], [58], [59]. Interestingly, we observed the greatest adaptation in muscle feeding (femoral) artery (Fig. 4D) whereas the endothelial mitochondrial content in the mesenteric artery was found even lower in VW than SED.

During exercise, the magnitude of WSS is increased to higher levels ranged from 15 to 30 dynes/cm^2^ in human arteries [29], [30], [33]. As an attempt to investigate underlying mechanisms of EC response to shear stress, and to better understand the effect of hemodynamics in endothelial/vascular health *in vivo*, several *in vitro* shear systems have been developed. Effects of the enhanced shear stress have been tested in numerous studies using an *in vitro* flow system, and these findings are consistent with those determined by *in vivo* studies [60]. In this study, we used 20 dyne/cm^2^ of high LSS as an exercise-mimicking flow condition, as it is within the range of arterial level shear stress [61].

In conclusion, our data support an idea that aerobic exercise enhances mitochondrial integrity in vascular endothelium which is essential for endothelial function. Shear stress seems to modulate signal transduction pathways towards mitochondrial biogenesis. Therefore, regulation on mitochondrial remodeling may represent one of the mechanisms whereby exercise-mediated increase in WSS confers a vasculoprotective effect. Future research is warranted to investigate the downstream and upstream of the shear-sensing mechanism and clinical implications of the shear stress-induced mitochondrial remodeling in preventing endothelial dysfunction.

## Supporting Information

Table S1 **LSS-Mediated Changes in Gene Expression of Glycolysis Markers.** Average fold changes of each of glycolysis markers are shown.
(DOCX)
