# Supplementary material for: Exercise-Mediated Wall Shear Stress Increases Mitochondrial Biogenesis in Vascular Endothelium
Source: PLoS One. 2014 Nov 6;9(11):e111409. doi: 10.1371/journal.pone.0111409 (PMC4222908; doi:10.1371/journal.pone.0111409)
Supplement: Table S1 — LSS-Mediated Changes in Gene Expression of Glycolysis Markers. Average fold changes of each of glycolysis markers are shown. (DOCX) [file pone.0111409.s001.docx]

**Table S1**

| **Name of Gene** | **Description** | **Fold Change** |
| --- | --- | --- |
| ALDOA | aldolase A, fructose-bisphosphate | -0.20 |
| ALDOB | aldolase B, fructose-bisphosphate | -0.14 |
| BPGM | 2,3-bisphosphoglycerate mutase | -0.85 |
| ENO1 | enolase 1, (alpha) | -0.79 |
| GAPDH | glyceraldehyde-3-phosphate dehydrogenase | -0.56 |
| GCK | glucokinase | -0.56 |
| GPI | glucose-6-phosphate isomerase | 0.07 |
| HK2 | hexokinase 2 | -1.27 |
| LDHA | lactate dehydrogenase A | -0.46 |
| LDHAL6A | lactate dehydrogenase A-like 6A | -0.26 |
| LDHAL6B | lactate dehydrogenase A-like 6B | 0.53 |
| LDHB | lactate dehydrogenase B | -0.66 |
| PFKFB1 | 6-phosphofructo-2-kinase/fructose-2,6-biphosphatase 1 | -0.91 |
| PFKFB2 | 6-phosphofructo-2-kinase/fructose-2,6-biphosphatase 2 | -0.49 |
| PFKP | phosphofructokinase, platelet | -1.10 |
| PGK1 | phosphoglycerate kinase 1 | -0.45 |
| PGK2 | phosphoglycerate kinase 2 | 0.03 |
| PGM1 | phosphoglucomutase 1 | -0.64 |
| PGM2 | phosphoglucomutase 2 | -0.16 |
| PGM3 | phosphoglucomutase 3 | -0.52 |
| TPI1 | triosephosphate isomerase 1 | -0.60 |
